# An importance-performance analysis of teachers’ perception of STEM engineering design education

**DOI:** 10.1057/s41599-023-01653-7

**Published:** 2023-04-10

**Authors:** Yu-Hung Chien, Fang-Yu Chang

**Affiliations:** 1 National Taiwan Normal University, Taipei, Taiwan; 2 Taichung Municipal Shengang Industrial High School, Taichung, Taiwan

**Keywords:** Education, Science, technology and society

## Abstract

There is an increasing worldwide trend toward the development of science, technology, engineering, and mathematics (STEM) education using engineering design (ED) practice. Considering that teachers play pivotal roles in terms of student interest in STEM subjects and careers, it is important to explore teachers’ perceptions of STEM-ED education. We analysed survey data from 184 technology teachers who participated in empowerment training (using a STEM-ED curriculum) in 2017, 2018, 2021, and 2022. We used an importance-performance analytical model to investigate the teachers’ perceptions of STEM-ED itself, its impact on students, and the challenges experienced during implementation. The results showed that various improvements were required for STEM-ED promotion and for the preparation of STEM-ED lessons. Analysis of variance revealed that the age groups taught and the number of weekly teaching hours significantly affected the teachers’ perceptions of STEM-ED. These findings will assist educational institutions worldwide in planning future education policies, designing teacher empowerment courses, and understanding teachers’ needs in efforts to improve STEM-ED.

## Introduction

To cultivate science, technology, engineering, and mathematics (STEM) competence, skills, and interest among students in the 21st century, governments worldwide have supported policy initiatives that promote STEM education with engineering design (ED) in secondary schools as a bridge to higher-level STEM fields (Chien and Chu, 2018; Cruz et al., 2021; Kutnick et al., 2020). This STEM-ED assists students in choosing future careers, motivates students to learn, and facilitates their interest in (and integration of) STEM subjects (Kwon, 2016).

In Taiwan, technology learning plays a pivotal role in STEM-ED for several reasons. First, technology is not a college entrance subject; technology teachers may be able to partially deviate from conventional test-oriented teaching methods (Asia Society, 2006) and thus contribute to an emerging pedagogy that promotes STEM-ED. Second, the Ministry of Education (MOE) published technology curriculum guidelines for K-12 students in 2014; these guidelines addressed engineering design, design thinking, and STEM (Fan and Yu 2017; MOE, 2018; Yu and Fan, 2017; Tsai et al., 2022), as well as the integration of interdisciplinary knowledge with project-based learning (Banks and Barlex, 2020; Fan and Yu, 2017; Yu and Fan, 2017). Third, most current technology teachers have science and technology qualifications; many have also been trained in engineering design education. Their basic content knowledge matches the STEM-ED curriculum (Fan and Yu, 2017; Yu and Fan, 2017).

Teaching competence is essential for the implementation and quality of STEM-ED (Honey et al. 2014); this competence was influenced by teachers’ learning experiences during their preparation programs (Berry et al. 2016; Hubers et al., 2022; Tsai et al., 2022). Although technology teachers may have prior training in engineering design, it remains challenging to familiarize these teachers with the goals of the new curriculum and to help them acquire the necessary pedagogical content knowledge (Yu and Fan, 2017; Tsai et al., 2022). Teacher professional development is needed to address these challenges (Barak, 2014). Therefore, we devised a STEM-ED course to provide basic content knowledge and pedagogical content knowledge for technology teachers, then investigated their views regarding STEM-ED by using an important-performance analysis (IPA) model (Martilla and James, 1977). The IPA was originally developed for marketing research; this model places mean scores of importance and performance in a two-dimensional matrix to analyse satisfaction and the importance of a product or service. The IPA has been used in education-related studies (Sever, 2015; Wohlfart et al., 2022). The IPA outcomes generated in the present study provide several practical suggestions, strategies, and methods that may assist future planning of educational policy worldwide; the results may also facilitate the design of teacher empowerment courses and support an understanding of teachers’ needs in efforts to improve STEM-ED. The empowerment course used in this study could serve as an example of teacher professional development that is applicable to STEM education worldwide.

## General background

### STEM-ED and empowerment training

STEM education enhances student knowledge, competence, and interest in STEM-related fields (NGSS Lead States, 2013); it also helps students to develop high-level thinking skills (Wells, 2016). The integration of engineering design into STEM education may also promote creativity and innovation (Land, 2013) while improving students’ abilities to understand complex technological and engineering concepts (Yu and Fan, 2017; Tsai et al. 2022). Previously, mathematics and science education focused on abstract theories rather than practical applications (Corum and Garofalo, 2015). STEM education may help students form associations between theory and practice (Chien and Chu, 2018), thus facilitating their future careers (Kwon, 2016).

There is a strong impetus for the integration of STEM education in Taiwan (MOE, 2018) and worldwide (Hubers et al., 2022; Kim and Bolger, 2017). The Taiwanese economy is based on science and technology; Taiwan is a “silicon island.” The semiconductor industry greatly contributes to Taiwan’s competitiveness; individuals with STEM talent are in high demand. Considering the nation’s complex economic and social contexts, the fertility rate is the lowest worldwide (Central Intelligence Agency, 2022); the number of university STEM graduates has significantly decreased (Taiwan News, 2020). Therefore, active cultivation of STEM education requires major policy efforts. Among these efforts, Taiwan’s new 12-year basic education program has prioritized STEM teaching within the technology education curriculum, which now plays a central role in teaching students to apply conceptual STEM knowledge to real-world challenges (MOE, 2018). Moreover, engineering design and project-based learning were incorporated into the technology curriculum such that it has evolved from a trial-and-error approach into a more systematic and logical approach to problem-solving (Yu and Fan, 2017). Students are expected to demonstrate technological literacy, hands-on abilities, an interest in course content, and problem-solving and creative thinking skills (Fan and Yu, 2017; Lee, 2019). However, studies of teachers’ opinions revealed that the teachers experienced difficulty with project-based inquiry methods; a new STEM curriculum was required (Liu et al., 2012). Therefore, empowerment training (a support measure) was needed (Barak, 2014; Lee, 2019; Yu and Fan, 2017) to help teachers achieve satisfactory STEM teaching outcomes (Honey et al., 2014). This training ensures that technology teachers’ professional credentials are adequate for the delivery of STEM-ED.

Previous studies have examined design-based STEM education (Guzey et al., 2016), along with its effects on decision-making (Altan et al., 2018), creativity (Hathcock et al., 2015; Altan and Tan, 2021), and STEM-related learning outcomes (Guzey et al., 2017). However, few studies have investigated teachers’ perceptions of STEM-ED. Other studies have investigated teachers’ perceptions and the implementation of STEAM (science, technology, engineering, arts, and mathematics) education (Park et al., 2016). Most teachers felt that STEAM education was essential for improving students’ motivation and learning; moreover, teachers desired help to overcome the challenges and difficulties associated with implementing STEAM education, including insufficient time and financial support to prepare lessons, insufficient teaching materials, and a lack of expertise (Han and Lee, 2012; Lee et al., 2013; Lim and Oh, 2015; Shin, 2013). Although these studies provided information concerning teachers’ perceptions and practices of STEAM education, they primarily included only elementary school teachers, and the design element was omitted. Consequently, there is an urgent need to investigate teachers’ perceptions of STEM-ED.

### IPA model

**Fig. 1:**
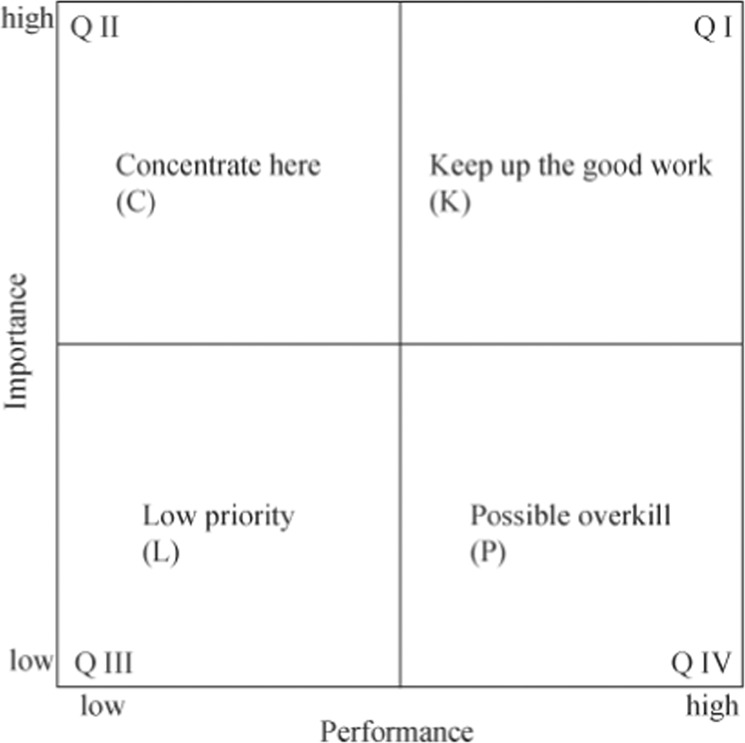
Representative of IPA graph (Martilla and James, 1977). The importance measure represents the vertical axis, and the performance measure constitutes the horizontal axis of a two-dimensional graph.

The IPA model analyses the attributes, strengths, and weaknesses of a product or a service, then identifies factors that require improvement (Martilla and James, 1977). Data analysis reveals four types of quality characteristics; this information facilitates the development of strategic actions that can enhance quality characteristics in each of the four quadrants. The model is then interpreted and the necessary strategic actions are formulated (Fig. 1).

IPA data are presented in *X*–*Y* coordinate charts (Martilla and James, 1977). The four quadrants (QI–QIV) reflect the mean amounts of performance and importance (Sever, 2015), whereas horizontal and vertical lines on the coordinate plane show the relationships between performance and importance. The interpretation of IPA matrixes involves the use of four Qs:

1. QI: Keep up the good work (K): The product or service quality characteristic is very important, and the organization performs well.
2. QII: Concentrate here (C): The product or service quality characteristic is very important, but the organization fails to perform.
3. QIII: Low priority (L): Although the product or service quality characteristic is not important, the organization fails to perform.
4. QIV: Possible overkill (P): Although the product or service quality characteristic is not important, the organization performs well.

Originally, the IPA was developed for marketing purposes, but it has since been applied in various fields, including education (Sever, 2015; Wohlfart et al., 2022). Previous educational studies used the IPA to focus on quality management and student satisfaction (Lakkoju, 2016) with curricula and facilities (McLeay et al., 2017); they also focused on teachers’ opinions about efforts to reform technology courses (Lin et al., 2015). Therefore, the IPA enables systematic and critical assessment of teachers’ perceptions regarding the implementation of STEM-ED in secondary schools; our research questions can be addressed. Using this approach, we can help to formulate educational policy and determine the practical needs of teachers who implement the curriculum. Based on the above considerations, the current study was guided by the following research questions:

RQ1: How do in-service technology teachers perceive STEM-ED overall?

RQ2: What background characteristics of in-service technology teachers affect their perceptions of STEM-ED?

RQ3: How do in-service technology teachers perceive the STEM-ED empowerment course?

## Methods

### Participants

We adopted the convenience sample method, including 184 technology teachers who enrolled in empowerment courses in 2017, 2018, 2021, and 2022, respectively. The empowerment course was not offered in 2019 and 2020 because of the coronavirus disease 2019 pandemic. The MOE entrusted higher education institutions with these empowerment courses for technology teachers willing to study further. Teachers who completed the course received a certificate of professional development.

### Design

We were commissioned by the MOE to develop a STEM-ED empowerment course for technology teachers. And as above-mentioned, the teachers enrolled in the courses between 2017 and 2022 were the participants of this study to provide their views regarding STEM-ED in a 19-item questionnaire and open-ended questions. The descriptive statistics and analysis of variance were used to analyse the collected data based on the IPA model. A detailed description of the development of the STEM-ED empowerment course, questionnaire, and data analysis were all in the following section.

### STEM-ED empowerment course

**Fig. 2:**
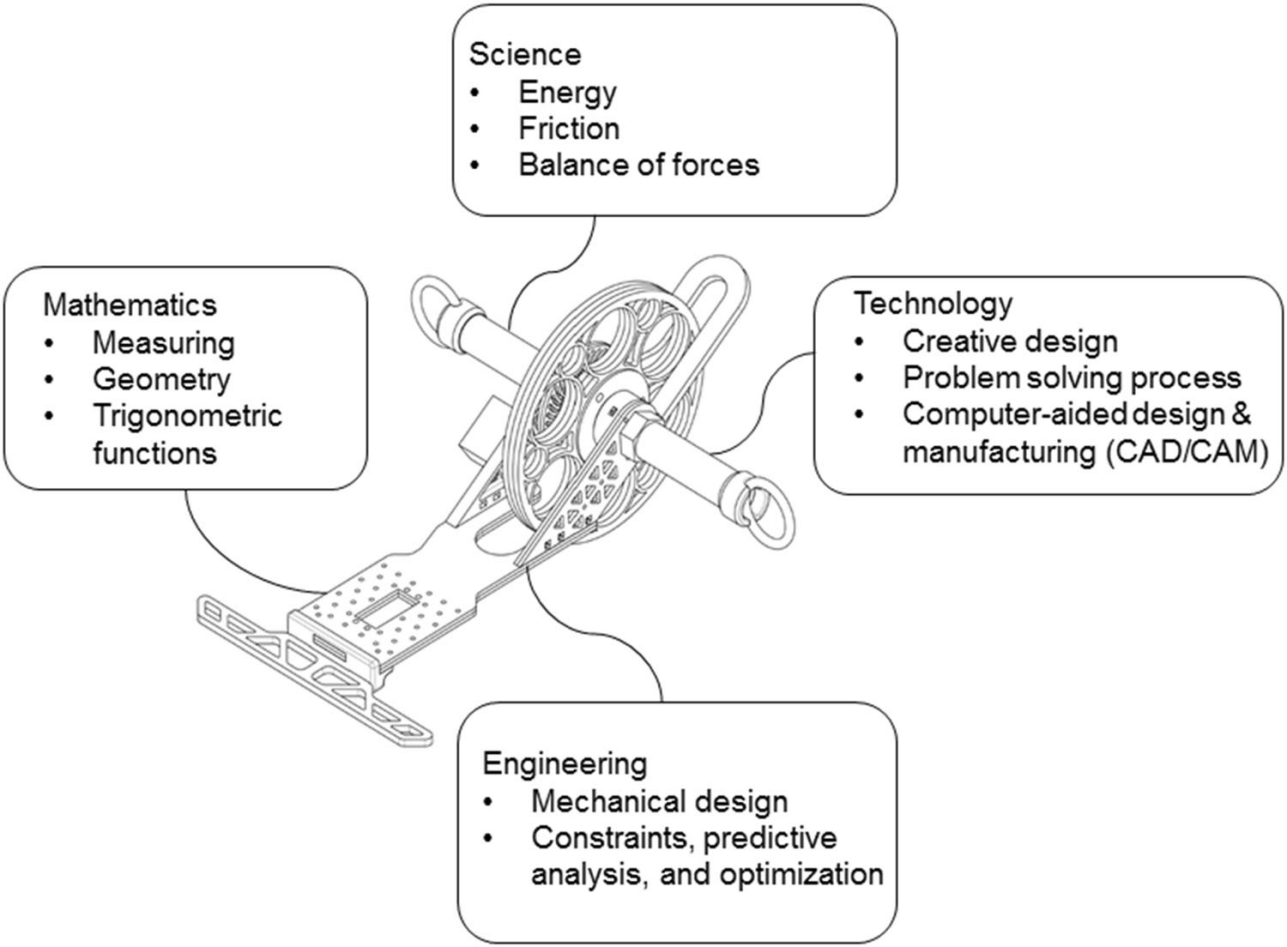
STEM knowledge covered by the empowerment course. Basic STEM knowledge was introduced in the course, and the technology teachers applied them during the engineering design process.

**Fig. 3:**
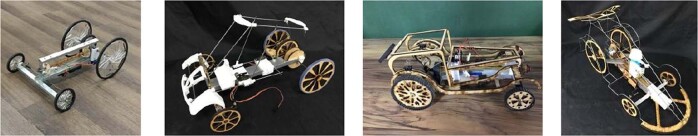
Teachers’ coursework. Technology teachers used STEM knowledge and steering and torque release strategies to design various elastic cars.

The STEM-ED course was inspired by the Transportation Design Department’s elastic formula racing car course at the Art Centre College of Design. The technology teachers were instructed to design a racing car and generate movement by twisting a rubber band (5 m long × 0.5 cm wide). They steered the cars around a track as quickly as possible using an infrared remote control and “torque release mechanism.” The course was based on the 10 design activities proposed by Atman (2019) related to energy conversion, friction, the balance between forces, mathematical measurements, geometry, and trigonometry. The teachers were expected to implement hands-on technological activities to promote artistic and creative car designs, emphasizing innovation and performance. The course length was 36 h over 5 days; the first 4 days were 8 h long, whereas day 5 was 4 h long. Table 1 is the detailed course contents, Fig. 2 shows the STEM knowledge covered by the empowerment course, and Fig. 3 shows the samples of the teachers’ coursework.

**Table 1:** Content of the 36-h STEM-ED empowerment course.

Day outline	Engineering design	Activities	Photos
Day 1: Introduce courses, hands-on activity, and STEM knowledge	• Identify need • Problem definition • Gather information	(1) Introduce computer-aided design and manufacturing. (2) Explain STEM-ED education. (3) Show videos of various elastic cars. (4) Introduce basic STEM knowledge used in this course. (5) Assemble basic mechanical parts of an elastic car.	
Day 2: Introduce basic mechanism design, 3D drawing application, and digital manufacturing tools	• Generate ideas • Communication • Decision • Modelling	(1) Introduce basic concepts of steering and torque release mechanism. (2) Learn 3D drawing. (3) Develop ideas of an elastic car. (4) Generate 3D drawing. (5) Learn digital machines (3D printer, laser cut machine, and CNC).	
Day 3: Make and test elastic car	• Modelling • Feasibility analysis • Evaluation • Decision	(1) Assemble and adjust the wheels of the elastic car. (2) Test the elastic car. (3) Redesign the elastic car.	
Day 4: Test elastic car and introduce teaching strategy	• Modelling • Feasibility analysis • Evaluation • Decision • Communication	(1) Test, Adjust, correct, optimize the elastic car. (2) Introduce teaching strategy of STEM-ED course. (3) Prepare presentation materials.	
Day 5: Present each team’s car design and perform the race activities.	• Communication • Implementation	(1) Present car design. (2) Perform car race activities. (3) Answer questionnaire.	

The course materials are available online (https://sites.google.com/ttsh.tp.edu.tw/rubber-band-car/).

After completing a 36-h STEM-ED empowerment course, each teacher completed the 19-item questionnaire and answered the open-ended questions, which required ~20 min. Teachers may discuss the open-ended questions.

### Measures

The measurement tool in this study was a 19-item questionnaire that included four questions regarding teachers’ background characteristics to determine whether teachers’ perceptions of STEM-ED differed according to age groups taught, teaching experience, number of weekly teaching hours in technology courses, and previous STEM training experience. Eleven IPA questions based on an earlier study by Park et al. (2016) [in accordance with the findings of a literature review (Han and Lee, 2012; Lee et al., 2013; Lim and Oh, 2015; Shin, 2013)] to investigate technology teachers’ perceptions of STEM-ED itself (questions 1–3); the impact of STEM-ED on students (questions 4–7); and challenges when implementing STEM-ED (questions 8–11). Four IPA questions related to the STEM-ED empowerment course (questions 12–15). The 15 IPA questions were 5-point Likert scale items. Moreover, an open-end question followed each aspect of the IPA questions to collect teachers’ explicit opinions in their own words.

The questionnaire was reviewed by five experts (three professors and two schoolteachers responsible for developing the empowerment course) to ensure that language, construct content, and face validity were acceptable. Fifteen teachers were invited to participate in a 6-h hands-on workshop regarding the empowerment course; they also participated in a pilot survey to validate the questionnaire. Feedback indicated that the questionnaire conformed to the practices and needs of teachers. The Cronbach alpha values of the four aspects of importance/performance were 0.75/0.70, 0.79/0.86, 0.62/0.70, and 0.70/0.73, respectively, indicating that the questionnaire exhibited acceptable internal consistency and reliability.

### Data analysis

Based on the IPA model, the mean values of performance (*x*-axis) and importance (*y*-axis) were used to establish two-dimensional matrices that yielded correlations indicated by the relative positions of the data points (Abalo et al., 2007; Martilla and James, 1977). Descriptive statistics were used to explore the teachers’ background characteristics. Analysis of variance was used to investigate whether teachers with different backgrounds had different views regarding performance assessment in terms of STEM-ED itself, the impact of STEM-ED on students, and the challenges involved in the implementation of STEM-ED. The results yielded insights into the most common priority rankings of various improvement strategies and directions in which to invest.

## Results

Based on the data from 184 questionnaires, we analysed teachers’ background characteristics and responses to the IPA questions. We also collected 95 answers (25 for the perception of STEM-ED itself, 17 for the impact of STEM-ED on students, 17 for challenges when implementing STEM-ED, and 36 for opinions regarding the STEM-ED empowerment course) from the teachers to the open-ended questions as qualitative evidence of the “lived” experience with quantitative data that would be described in the Discussion.

### Analysis of teachers’ background characteristics

Taiwan has 1211 certified middle school technology teachers, which is approximately 1.5-fold greater than the number of high school technology teachers (*N* = 954). The MOE designated teams from three universities to provide empowerment courses for these technology teachers. Thus far, 465 teachers have participated in these courses. We collected data from 184 teachers who participated in the empowerment course offered by the authors’ team from 2017 to 2022. Table 2 lists the teachers’ background characteristics.

**Table 2:** Background characteristics analysis (*N* = 184).

Background characteristics		*N*	%
Age groups taught	Middle school	141	76.6
	High school	26	14.1
	Combined	17	9.2
Teaching experience	1–5	32	17.4
	6–10	23	12.5
	11–15	33	17.9
	16–20	39	21.2
	21 & more	57	31.0
Number of weekly teaching hours	0	43	23.4
	1–17	90	48.9
	18 & more	51	27.7
STEM training experience	Yes	73	39.7
	No	111	60.3

Of the 184 teachers in this study, 141 were middle school teachers (5.42-fold greater than the number of high school teachers; *N* = 26). Furthermore, 52.2% were senior teachers (>15 years of teaching experience), 78.3% did not teach sufficient hours to meet the standard teaching hours (18 h per week), 43% did not teach any technology course, and 39.6% had previous STEM-related training experience.

### IPA question analysis

Table 3 shows the total mean scores for the perception of STEM-ED itself, the impact of STEM-ED on students, challenges when implementing STEM-ED, and opinions regarding the STEM-ED empowerment course. The four mean scores for the importance levels were all >4. Among the four mean scores of performance assessment, only “challenges when implementing STEM-ED” was <4 (mean = 3.75, standard deviation = 0.66). Among the 15 IPA questions, seven were in QI (K), two (1 and 9) were in QII (C), five were in QIII (L), and one was in QIV (P).

**Table 3:** Analysis results of the IPA questions.

Aspects/Questions	Mean (SD)		IPA quadrant/category
	Importance	Performance	
*The perception of STEM-ED itself*	4.45(0.53)	4.32(0.59)	
1. Promoting STEM-ED to students’ interest and future talents in STEM fields.	4.61(0.49)	4.29(0.76)	II/C
2. Recommendation of STEM-ED to colleagues.	4.42(0.73)	4.40(0.73)	IV/P
3. Implementation of STEM-ED courses in teaching.	4.33(0.71)	4.26(0.74)	III/L
*The impact of STEM-ED on students*	4.36(0.46)	4.27(0.63)	
4. Students’ critical thinking.	4.42(0.50)	4.28(0.77)	I/K
5. Students’ creativity.	4.56(0.59)	4.37(0.74)	I/K
6. Students’ personal traits.	4.09(0.68)	4.09(0.80)	III/L
7. Students’ choices in STEM disciplines.	4.37(0.58)	4.28(0.77)	I/K
*Challenges in implementing STEM-ED*	4.68(0.32)	3.75(0.66)	
8. Support of administration and finance.	4.85(0.36)	3.87(0.94)	I/K
9. Readiness of STEM-ED lessons.	4.75(0.48)	3.76(0.87)	II/C
10. Workload	4.57(0.50)	3.68(0.88)	III/L
11. Use of new equipment and media.	4.59(0.55)	3.67(0.94)	III/L
*Opinions of the STEM-ED empowerment course*	4.52(0.40)	4.48(0.49)	
12. Provision of empowerment training in STEM-ED teaching strategy.	4.62(0.49)	4.49(0.60)	I/K
13. Promotion of empowerment training.	4.53(0.50)	4.58(0.66)	I/K
14. Appropriate content of empowerment training.	4.57(0.50)	4.49(0.65)	I/K
15. Length of empowerment training.	4.35(0.70)	4.36(0.75)	III/L

Table 4 shows the results of the analysis of variance according to the teachers’ background characteristics. The age groups taught significantly affected the performance assessment scores on “the perception of STEM-ED itself” [*F*(2180) = 4.38, *p* = 0.02, *ηp*^2^ = 0.05] and “opinions regarding the STEM-ED empowerment course” [*F*(2180) = 4.06, *p* = 0.05, *ηp*^2^ = 0.03]. The number of weekly teaching hours significantly affected the performance assessment scores for “challenges when implementing STEM-ED” [*F*(2180) = 4.05, *p* = 0.02, *ηp*^2^ = 0.04].

**Table 4:** Summary table of the analysis of variance.

Aspects	IPA Model	*F*-value (LSD)			
		Age groups taught	Year of teaching	Number of weekly teaching hours	STEM training
The perception of STEM-ED itself	Importance	1.83	0.75	0.12	0.37
	Performance	4.38*(3 < 1)	1.61	0.26	0.28
The impact of STEM-ED on students	Importance	0.43	0.60	0.35	0.14
	Performance	1.34	0.50	0.41	0.12
Challenges in implementing STEM-ED	Importance	0.09	1.51	1.30	0.20
	Performance	0.97	0.34	4.05*(3 < 1)	0.54
Opinions of the STEM-ED empowerment course	Importance	0.49	0.85	0.44	0.27
	Performance	4.06*(3 < 1.2)	1.10	0.58	0.35

^*^ *p* < 0.05.

**Fig. 4:**
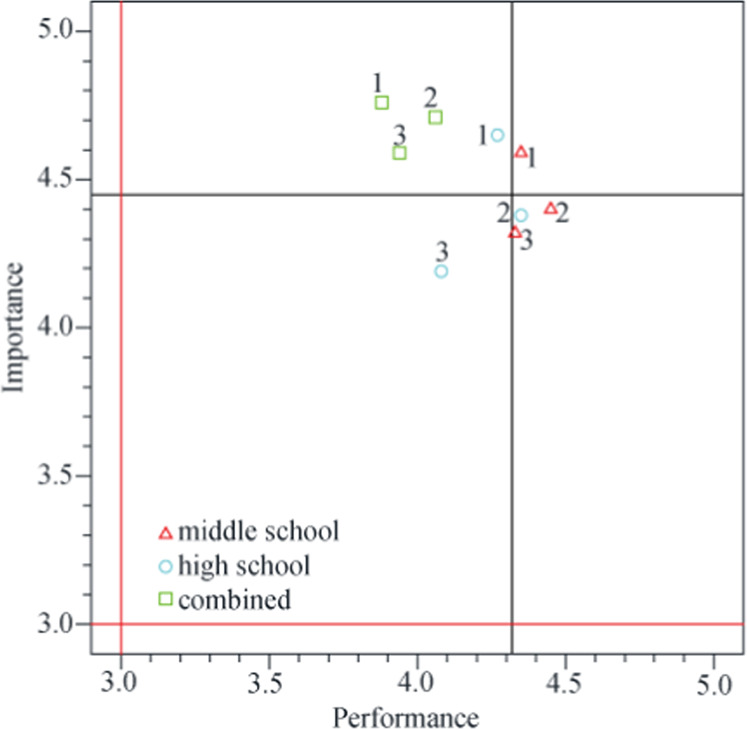
IPA values of “the perception of STEM-ED itself” in the different age groups taught. The performance scores (green squares) of the combined group of teachers were all located in QII.

Of the three IPA questions in “the perception of STEM-ED itself” (Table 3), question 1 was categorized in QII (C), question 2 was categorized in QIV (P), and question 3 was categorized in QIII (L). According to the IPA model (Martilla and James, 1977), question 1 (i.e., promotion of STEM-ED to colleagues and students to foster the students’ interests and future talents in STEM fields) requires priority attention. Moreover, the age groups taught significantly affected the assessment of performance levels. Figure 4 shows the significant differences in IPA values. The performance scores of teachers in the combined group were below the mean value (mean = 4.32, standard deviation = 0.59), whereas the scores of middle school teachers were all above the mean value.

“The impact of STEM-ED on students” included four IPA questions. Questions 4, 5, and 7 were categorized in QI (K); question 6 was categorized in QIII (L) (Table 3). There were no significant differences among teacher groups.

“Challenges when implementing STEM-ED” also included four IPA questions. As shown in Table 3, question 8 was categorized in QI (K), question 9 was categorized in QII (C), and questions 10 and 11 were categorized in QIII (L). Question 9 (preparation of STEM-ED lessons) requires priority care (Martilla and James, 1977).

**Fig. 5:**
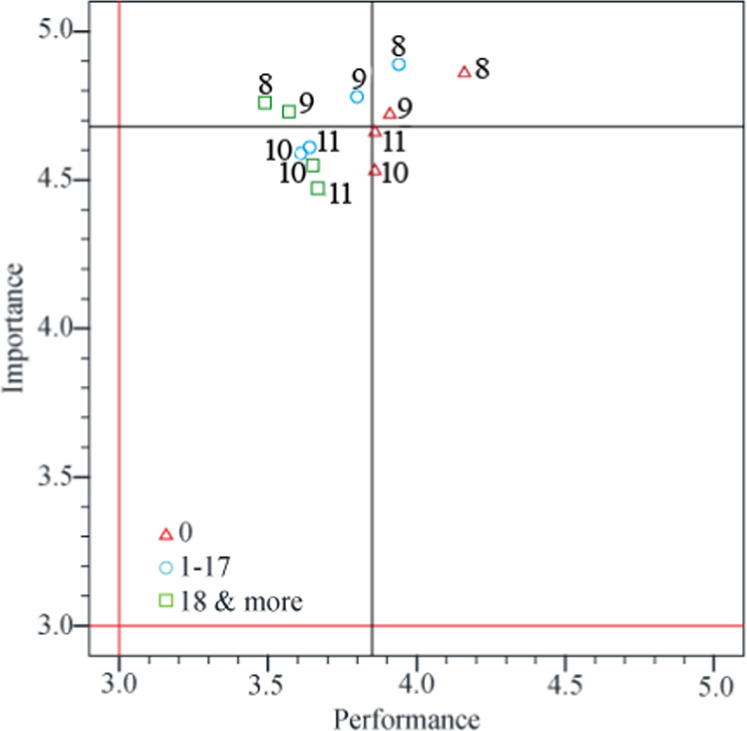
IPA values of “challenges when implementing STEM-ED” in the different number of weekly teaching hours. All four green squares (representing teachers who taught ≥18 h per week) were on the mean score line’s lower score side.

Figure 5 shows the significant differences in IPA values. The assessment of performance levels significantly differed according to the number of weekly teaching hours. Teachers who taught ≥18 h per week had scores below the mean on all four IPA questions (mean = 3.75, standard deviation = 0.66), whereas teachers who taught 0 h per week had scores above the mean on all four IPA questions.

**Fig. 6:**
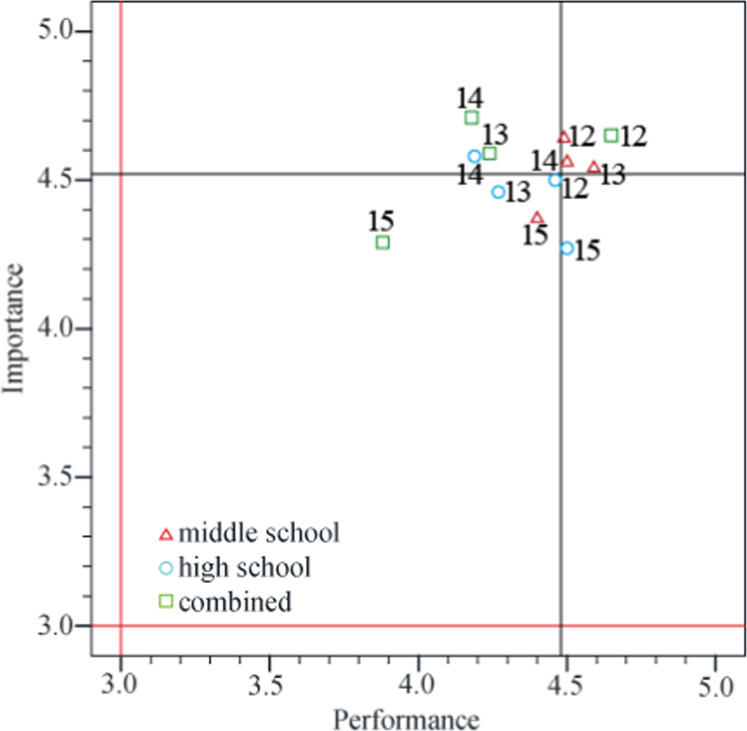
IPA values of “opinions regarding the STEM-ED empowerment course” the different age groups taught. The mean score on question 15 among teachers in the combined group is much lower than ≪ the other scores.

“Opinions regarding the STEM-ED empowerment course” included four IPA questions. Table 3 shows that questions 12–14 were categorized in QI (K) and question 15 was categorized in QIII (L). Figure 6 shows the significantly different values, which mainly arose from the lower mean score on question 15 among teachers in the combined group.

## Discussion

### RQ1: How do in-service technology teachers perceive STEM-ED overall?

The mean scores of the importance of the perception of STEM-ED itself, the impact of STEM-ED on students, and challenges when implementing STEM-ED were all >4, indicating that most teachers agreed with the importance of the three aspects. In terms of performance assessment, only “challenges when implementing STEM-ED” was <4. Thus, teachers demonstrated a need to consider teacher workload when developing STEM-ED, but they currently do not feel challenged. This finding differs from the results of previous studies (Han and Lee, 2012; Lee et al., 2013; Lim and Oh, 2015; Shin, 2013) and will be discussed in greater detail below.

The IPA model (Martilla and James, 1977) suggests that IPA values in QII (i.e., questions 1 and 9) should be prioritized. The teachers felt that the promotion of STEM-ED was important, but the current status could be improved; they reported some weaknesses in terms of their ability to prepare STEM-ED lessons. Recent research has revealed various methods by which STEM-ED can be promoted during teacher training. For example, leading teachers can perform curriculum analyses that include various best practices when aiding STEM-ED learning (Aykan and Yıldırım, 2022); this approach helps new teachers to develop skills and improve their lesson planning (Fernandez, 2010). Research-based pedagogies are incorporated into teacher education to promote knowledge regarding STEM-ED teaching and a mindset that encourages growth (Milner-Bolotin, 2018).

For “the perceptions of STEM-ED itself”, teachers generally had a positive perception of STEM-ED itself, consistent with the findings in previous STEAM education studies (Han and Lee, 2012; Lim and Oh, 2015; Park et al., 2016; Shin, 2013; Shin and Han, 2011). However, the implementation of STEM-ED courses can be further refined. The assessment score was significantly lower for teachers in the combined group than for middle school teachers. Teachers of both middle and high school students would likely consider curriculum design from the perspective of continuity and differences between age groups; they may also perceive that the provision of STEM-ED to different age groups is more challenging. According to the answers to the open-ended question, several teachers made statements similar to the following:

When teaching middle and high school students concurrently, we need more help to prepare new courses appropriate for the core literacy and learning foci of the different age groups. Otherwise, we will be teaching the same things to students at different levels.

Middle and high school students have different teaching goals and needs, according to the curriculum guidelines (Yu and Fan, 2017). Thus, teachers have considerable responsibility to design a curriculum for students at different learning stages. Accordingly, empowerment training should provide teachers with the pedagogical content knowledge required for STEM-ED, while considering the difficulties and challenges experienced by teachers. Various tools should be used to assist teachers who work with multiple age groups (Lee, 2019; Yu and Fan, 2017).

For “the impact of STEM-ED on students”, according to scores on the four relevant IPA questions, teachers felt that STEM-ED had a positive impact on analytical thinking, creativity, and personal traits among students, as well as the students’ future selection of a STEM career. These findings were consistent with the results of previous studies, where the cross-domain knowledge obtained through STEM/STEAM education promoted analytical thinking among students, while STEM-ED inspired creativity. Cross-domain knowledge helps students to identify their strengths, improve personal traits, and choose relevant academic fields; ultimately, this knowledge helps to cultivate STEM talent (Han and Lee, 2012; Kim and Bolger, 2017; Lim and Oh, 2015; Park et al., 2016; Shin and Han, 2011). However, one of the teachers in this study mentioned the following:

STEM-ED requires more input from students. Actually, this type of teaching strategy tests teachers’ teaching ability even more than before, since students spend most of their time studying admission exam subjects, with limited time available for non-exam subjects.

Teacher efficacy in terms of STEM education is dependent upon teachers’ knowledge of teaching (National Research Council, 2014) and is related to student persistence with, and retention in, STEM subjects (Painter and Bates, 2012). Under the credential system in Asian countries, the degree to which students can devote themselves to technology courses is affected by the prioritization of examination subjects. Thus, there is a need to develop various teaching strategies to ensure student persistence with, and retention in, the STEM-ED curriculum (Lee et al., 2004; Lin et al., 2015). The learning process of a STEM-ED course must enable students to explore other academic directions, gain a sense of accomplishment during the learning process through diversified learning, and apply the acquired knowledge to real-life problems. In this manner, schools, parents, and students will view STEM-ED as a means to enhance students’ talents (Altan et al., 2018; Altan and Tan, 2021).

For “challenges in the implementation of STEM-ED”, it is the only aspect for which the mean scores of the four IPA questions were all <4. Although the mean scores of the four IPA questions were lower than the mean scores of other dimensions, the IPA graph showed that teachers performed appropriately when they were supported by school administration and finance departments. The teachers had minimal concerns about the teaching load and any new equipment or media. This finding differs from the results of previous studies involving participants from various teaching fields, rather than technology alone (Park et al., 2016). Intriguingly, teachers with high teaching loads per week were particularly capable of implementing STEM-ED. One teacher stated:

The high teaching load for technology subjects can help teachers be more flexible when preparing teaching materials and courses, such as machinery and computers, and technology and computer classrooms.

Because technology is a new educational focus, the MOE has recently invested more funds and resources in establishing technology education centres and creative laboratories nationwide (Lee, 2019). Moreover, the MOE funds the purchase and maintenance of digital tools and equipment (e.g., laser cutting machinery, 3-D printers, Arduino microprocessors, and robots) to ensure efficient course implementation (Chien and Chu, 2018). Accordingly, teachers may perceive that administrative and financial support is appropriate, easing the teaching load. However, question 9 (ability to prepare STEM-ED lessons) requires attention (as revealed by the IPA model; Martilla and James, 1977). Therefore, in addition to hardware resources, teachers require professional development that enhances their knowledge, skills, and dispositions in STEM-ED, thus empowering them to provide quality STEM-ED (Cotabish et al., 2011).

### RQ2: What background characteristics of in-service technology teachers affect their perceptions of STEM-ED?

The teachers’ background characteristics provide some insights into the current status of STEM-ED development. The number of middle school teachers enrolled in the empowerment courses was approximately 5-fold greater than that of high school teachers. Middle school teachers have more urgent needs in terms of STEM-ED empowerment training because middle schools provide 3-year compulsory STEM courses that become elective after the 10th grade (MOE, 2018). Teachers weighed the priorities of their teaching responsibilities and participated in empowerment training to meet the guidelines of the new education curriculum.

More than half of the teachers overall had ≥15 years of teaching experience. This reflects the importance of (and strong demand for) senior teachers when new educational policies and teaching content are established; such teachers were willing to participate in STEM-ED. One senior teacher stated:

I believe that most technology teachers can actively participate in, understand, and adapt to teaching new things, and so understand and agree with new education strategies. We did not learn enough in school; now we have to learn a lot of new teaching methods. As new developments emerge, technology teachers should keep their knowledge up-to-date.

Furthermore, 43 technology teachers did not actually teach technology courses. In the previous curriculum, physics, chemistry, biology, earth science, and technology were all in the same learning area. Schools were free to allocate time to these subjects within the same field. Technology is not a university entrance subject; schools tend to prioritize examination subjects (Lee, 2019). Consequently, technology teachers often switch to other subjects or assume administrative duties (Lin, 2007), either voluntarily or because such a change is required. In the new curriculum guidelines, technology is separated from science (Fang, 2019; Lee, 2019; Ritz and Fan, 2015; Yu and Fan, 2017). Thus, there is an urgent need for teachers to promote the new technology curriculum, and many technology teachers are willing to return to technology (Lee, 2019); these teachers attended the empowerment courses. One senior technology teacher stated:

We are pleased that technology has been separated from science as a stand-alone learning area, which guarantees teaching time for technology. We are willing to participate in the empowerment training and prepare for the new teaching guidelines.

### RQ3: How do in-service technology teachers perceive the STEM-ED empowerment course?

The scores of the four IPA questions 12–15 reflected positive teacher attitudes toward the empowerment course. However, scores on the “length of empowerment training” significantly varied according to the age groups taught. Several responses from teachers in the combined group were notable, including the following:

Although there was much to be gained from the 8-hour per day training week, the course content was challenging to retain and apply, regardless of the basic content knowledge and the necessary pedagogical content knowledge.

Teachers in the combined group felt that the knowledge obtained from the empowerment course could not be directly applied; moreover, the basic content knowledge may be inappropriate for teaching at middle schools versus high schools.

The provision of STEM education training to teachers would increase their STEM knowledge and confidence with respect to teaching STEM (Nadelson et al., 2012). Clear educational goals and systematic planning of the empowerment curriculum, considering core literacy and learning foci, are needed to promote professional development programs for STEM-ED (Tsai et al., 2022). The current empowerment course requires greater emphasis on systematic planning and clear teaching goals, enabling teachers to implement courses within their schools for students in different learning stages.

Finally, we did not offer empowerment courses in 2019 and 2020 because of the difficulties of online access to tools, materials, and resources for hands-on learning; we suspected that teachers’ motivation and engagement might be affected by these difficulties (Code et al., 2020). However, hands-on technology education informed by a pandemic-transformed pedagogy is possible (Wright and Bartholomew, 2020); such education may be essential for maintaining the motivation and opportunities needed for teacher empowerment training and the resultant high-quality student learning.

## Conclusions

Considering the increasing worldwide dependence on STEM-related knowledge and skills (Sen et al., 2018), STEM-ED plays a key role in fostering positive dispositions toward STEM (Cotabish et al., 2011). Teachers must accept new STEM/STEAM teaching strategies and understand how to successfully implement STEM-ED. Additionally, it is important to assess their opinions of this new teaching strategy. The results of this study support the following conclusions related to the future of STEM-ED.

First, the technology teachers had a positive attitude toward promoting STEM-ED. Technology teachers already have STEM-related knowledge and can adapt to emerging educational approaches. They also expressed willingness to attend relevant courses to keep up-to-date with curriculum developments, and they stated that STEM-ED would help improve students’ learning motivation, thinking skills, and interest in pursuing STEM subjects. Second, the technology teachers were not concerned about the additional workload that STEM-ED would entail, but they were concerned that the new course content may require greater input from students. Therefore, strategies are needed to enhance the recognition of STEM-ED by students, parents, and schools, with the aim of encouraging students to engage with STEM/STEAM learning (Herro and Quigley, 2017). Third, professional development programs should use various methods and include diverse content to help teachers meet the core literacy and learning foci requirements when designing and implementing the STEM-ED curriculum. Professional development programs should also help teachers to address any teaching-related problems that they may experience (Lin et al., 2015). Finally, the IPA model (Martilla and James, 1977) shows that, of the various improvements required, the most important is the promotion of STEM-ED and the ease of STEM-ED lesson preparation.

This study has some limitations that should be addressed in further possible studies by STEM education researchers from various fields. First, only 465 technology teachers have participated in the formal empowerment training provided by the MOE. We collected data from 184 of these 465 teachers (~40%) from 2017 to 2022. We believe that the sample size in this study was adequate and that the multi-year nature of the study provided important insights into methods that can improve the professional development program for STEM-ED empowerment training. Most teachers who participated in this study had voluntarily enrolled in the empowerment training, suggesting that they were motivated and open to learning about new pedagogical approaches. However, such motivation may not persist among technology teachers if the empowerment courses become mandatory. Finally, studies with more diverse samples of teachers are needed. Second, this multi-year study provided critical insights into teachers’ perceptions of STEM-ED, and the findings will be useful in the improvement and expansion of STEM-ED professional development programs. The next step in the establishment of such programs should involve an assessment of the effectiveness of STEM-ED units taught in the classroom. Third, the 19-item questionnaire used in this study was based on a small-scale population and focused on teachers’ perceptions of engineering-based STEM education. Future research can develop formal scales for a general STEM education to investigate teachers’ and students’ metacognition of curriculum, self-efficiency, and other issues to prepare for the possibility of large-scale and extensive implementation of STEM education.

## Data Availability

The datasets generated during and/or analysed during the current study are available from the corresponding author upon reasonable request.

## References

[CR1] Abalo J, Varela J, Manzano V (2007). Importance values for importance-performance analysis: a formula for spreading out values derived from preference rankings. J Bus Res.

[CR2] Altan EB, Tan S (2021). Concepts of creativity in design based learning in STEM education. Int J Technol Des Educ.

[CR3] Altan EB, Yamak H, Kirikkaya EB, Kavak N (2018). The use of design-based learning for STEM education and its effectiveness on decision making skills. Univ J Educ Res.

[CR4] Asia Society (2006) Math and science education in a global age: what the U.S. can learn from China. http://www.asiasociety.org/files/math-science-china.pdf. Accessed 21 Jan 2023

[CR5] Atman CJ (2019). Design timelines: concrete and sticky representations of design process expertise. Des Stud.

[CR6] Aykan A, Yıldırım B (2022). The integration of a lesson study model into distance STEM education during the covid-19 pandemic: teachers’ views and practice. Technol Knowl Learn.

[CR7] Banks F, Barlex D (2020) Teaching STEM in the secondary school: helping teachers meet the challenge. Routledge

[CR8] Barak M (2014). Closing the gap between attitudes and perceptions about ICT-enhanced learning among pre-service STEM teachers. J Sci Educ Technol.

[CR9] Berry A, Depaepe F, Driel J, Loughran J, Hamilton M (2016). Pedagogical content knowledge in teacher education. International handbook of teacher education.

[CR10] Central Intelligence Agent (2022) Country comparisons birth rate. https://www.cia.gov/the-world-factbook/field/birth-rate/country-comparison/. Accessed 21 Jan 2023

[CR11] Chien YH, Chu PY (2018). The different learning outcomes of high school and college students on a 3D-printing STEAM engineering design curriculum. Int J Sci Math Educ.

[CR12] Code J, Ralph R, Forde K (2020). Pandemic designs for the future: perspectives of technology education teachers during COVID-19. Inf Learn Sci.

[CR13] Corum K, Garofalo J (2015). Using digital fabrication to support student learning. 3D Print Addit Manuf.

[CR14] Cotabish A, Dailey D, Hughes GD, Robinson A (2011). The effects of a STEM professional development intervention on elementary teachers’ science process skills. Res Sch.

[CR15] Cruz J, Bruhis N, Kellam N, Jayasuriya S (2021). Students’ implicit epistemologies when working at the intersection of engineering and the arts. Int J STEM Educ.

[CR16] Fan SC, Yu KC (2017). How an integrative STEM curriculum can benefit students in engineering design practices. Int J Technol Des Educ.

[CR17] Fang DL (2019). An inquiry into the development and review process of the 12-year basic education curriculum guidelines [in Chinese with English abstract]. J Educ Res.

[CR18] Fernandez ML (2010). Investigating how and what prospective teachers learn through microteaching lesson study. Teach Teach Educ.

[CR19] Guzey SS, Moore TJ, Harwell M (2016). Building up STEM: an analysis of teacher-developed engineering design-based STEM integration curricular materials. J Pre-Coll Eng Educ Res.

[CR20] Guzey SS, Harwell M, Moreno M, Peralta Y, Moore TJ (2017). The impact of design-based STEM integration curricula on student achievement in engineering, science, and mathematics. J Sci Educ Technol.

[CR21] Han H, Lee H (2012). A study on the teachers’ perceptions and needs of STEAM education. J Learn-Cent Curric Instr.

[CR22] Hathcock SJ, Dickerson DL, Eckhoff A, Katsioloudis P (2015). Scaffolding for creative product possibilities in a design-based STEM activity. Res Sci Educ.

[CR23] Herro D, Quigley C (2017). Exploring teachers’ perceptions of STEAM teaching through professional development: Implications for teacher educators. Prof Dev Educ.

[CR24] Honey M, Pearson G, Schweingruber H (2014) STEM integration in K-12 education: Status, prospects, and an agenda for research. The National Academies Press

[CR25] Hubers MD, Endedijk MD, van Veen K (2022). Effective characteristics of professional development programs for science and technology education. Prof Dev Educ.

[CR26] Kim D, Bolger M (2017). Analysis of Korean elementary pre-service teachers’ changing attitudes about integrated STEAM pedagogy through developing lesson plans. Int J Sci Math Educ.

[CR27] Kutnick P, Lee BPY, Chan RYY, Chan CKY (2020). Students’ engineering experience and aspirations within STEM education in Hong Kong secondary schools. Int J Educ Res.

[CR28] Kwon H (2016). Effect of middle school students’ motivation to learn technology on their attitudes toward engineering. Eurasia J Math Sci Technol Educ.

[CR29] Lakkoju S (2016). Importance-performance analysis of service quality in higher education: a case study. Indian J Ind Relat.

[CR30] Land MH (2013). Full STEAM ahead: the benefits of integrating the arts into STEM. Procedia Comput Sci.

[CR31] Lee JW, Park HJ, Kim JB (2013). Primary teachers’ perception analysis on development and application of STEAM education program. J Korean Soc Elem Sci Educ.

[CR32] Lee LS (2019) Status and challenges of the technology education as an integral part of general education in Taiwan. Paper presented at the 13th international conference on technology education in the Asia Pacific Region (2019 ICTE-Korea), Cheongju, South Korea, 16–18 Jan 2019

[CR33] Lee LS, Wang ST, Wang B, Ko J, Wang CH, He CC, Wu HL et al (2004) A brief introduction to technology education in Taiwan. https://files.eric.ed.gov/fulltext/ED507763.pdf. Accessed 21 Jan 2023

[CR34] Lim CH, Oh BJ (2015). Elementary pre-service teachers and in-service teachers’ perceptions and demands on STEAM education [in Korean with English abstract]. J Korean Soc Earth Sci Educ.

[CR35] Lin KY (2007). Ethical issues in technology education in Taiwan. J Technol Stud.

[CR36] Lin KY, Chang LT, Tsai FH, Kao CP (2015). Examining the gap between teaching and learning in the technology curriculum within Taiwan’s 9-year articulated curriculum reform from the perspective of curriculum implementation. Int J Technol Des Educ.

[CR37] Liu X, Liang LL, Liu E (2012). Science education research in China: challenges and promises. Int J Sci Educ.

[CR38] Martilla JA, James JC (1977). Importance-performance analysis. J Mark.

[CR39] McLeay F, Robson A, Yusoff M (2017). New applications for importance-performance analysis (IPA) in higher education: understanding student satisfaction. J Manag Dev.

[CR40] Milner-Bolotin M (2018). Evidence-based research in STEM teacher education: from theory to practice. Front Educ.

[CR41] Ministry of Education (MOE) (2018) The technology learning area curriculum guidelines in the 12-year compulsory education. https://www.naer.edu.tw/ezfiles/0/1000/attach/52/pta_18529_8438379_60115.pdf. Accessed 21 Jan 2023

[CR42] Nadelson LS, Seifert A, Moll AJ, Coats B (2012). i-STEM summer institute: an integrated approach to teacher professional development in STEM. J STEM Educ.

[CR43] National Research Council (2014) STEM integration in K-12 education: status, prospects, and an agenda for research. National Academies Press

[CR44] NGSS Lead States (2013) Next generation science standards: for states, by states. The National Academies Press

[CR45] Painter P, Bates R (2012). Statistical models of self-efficacy in STEM students. J Undergrad Res.

[CR46] Park H, Byun SY, Sim J, Han HS, Baek YS (2016). Teachers’ perceptions and practices of STEAM education in South Korea. Eurasia J Math Sci Technol Educ.

[CR47] Ritz JM, Fan SC (2015). STEM and technology education: International state-of-the-art. Int J Technol Des Educ.

[CR48] Sen C, Ay ZS, Kiray SA, Shelly M, Kiray SA (2018). STEM skills in the 21st century education. Research highlights in STEM education.

[CR49] Sever I (2015). Importance–performance analysis: a valid management tool?. Tour Manag.

[CR50] Shin JH (2013). Survey of primary & secondary school teachers’ recognition about STEAM convergence education [in Korean with English abstract]. Korean J Learn Sci.

[CR51] Shin Y, Han S (2011). A study of the elementary school teachers‘ perception in STEAM (Science, Technology, Engineering, Arts, Mathematics) education [in Korean with English abstract]. J Korean Elem Sci Educ.

[CR52] Taiwan News (2020) Taiwan mulls loosening labour rules to allow more foreign IT graduates. https://www.taiwannews.com.tw/en/news/4038503. Accessed 21 Jan 2023

[CR53] Tsai FH, Hsiao HS, Yu KC, Lin KY (2022). Development and effectiveness evaluation of a STEM-based game-design project for preservice primary teacher education. Int J Technol Des Educ.

[CR54] Wells JG (2016). Efficacy of the technological/engineering design approach: imposed cognitive demands within design-based biotechnology instruction. J Technol Educ.

[CR55] Wohlfart O, Adam S, Hovemann G (2022). Aligning competence-oriented qualifications in sport management higher education with industry requirements: an importance-performance analysis. Ind High Educ.

[CR56] Wright GA, Bartholomew SR (2020). Hands-on approaches to education during a pandemic. Technol Eng Teach.

[CR57] Yu KC, Fan SC (2017) The development of new technology teacher education curriculum in Taiwan. Paper presented at the TENZ-ICTE Conference, St Margaret’s College, New Zealand, 8–11 Oct 2017

